# Sinapicacid Inhibits Group IIA Secretory Phospholipase A_2_ and Its Inflammatory Response in Mice

**DOI:** 10.3390/antiox11071251

**Published:** 2022-06-25

**Authors:** Aladahalli S. Giresha, Deepadarshan Urs, Sophiya Pundalik, Rajkumar S. Meti, Siddanakoppalu N. Pramod, Ballenahalli H. Supreetha, Madhusudana Somegowda, Kattepura K. Dharmappa, Ahmed M. El-Shehawi, Sarah Albogami, Mona M. Elseehy, Abdullah Alaklabi, Hosam O. Elansary, Alanoud Omur A. Mehder, Eman A. Mahmoud

**Affiliations:** 1 Inflammation Research Laboratory, Department of Studies and Research in Biochemistry, Mangalore University, Jnana Kaveri Post Graduate Campus, Chikka Aluvara, Kodagu 571232, India; girishas25@mangaloreuniversity.ac.in (A.S.G.); deepdarshanurs@mangaloreuniversity.ac.in (D.U.); p.sophiya89@mangaloreuniversity.ac.in (S.P.); rsmeti@gmail.com (R.S.M.); suprithabh5985@gmail.com (B.H.S.); 2 Department of Biochemistry, School of Science, Jain (Deemed-to-Be University), JC Road, Bangalore 560027, India; 3 Department of Studies in Food Technology, Davangere University, Shivagangothri, Davangere 577007, India; pramodsn@davangereuniversity.ac.in; 4 Department of Plant Biochemistry, University of Agriculture and Horticulture Science, Shivamogga 577204, India; ysmadhu84@gmail.com; 5 Department of Biotechnology, College of Science, Taif University, Taif 21944, Saudi Arabia; a.elshehawi@tu.edu.sa (A.M.E.-S.); dr.sarah@tu.edu.sa (S.A.); 6 Department of Genetics, Faculty of Agriculture, University of Alexandria, Alexandria 21545, Egypt; monaahmedma@yahoo.com; 7 Department of Biology, Faculty of Science, University of Bisha, Bisha 61922, Saudi Arabia; alaklabia@gmail.com; 8 Plant Production Department, College of Food & Agriculture Sciences, King Saud University, Riyadh 11451, Saudi Arabia; 9 Family Education Department, Education College, Umm AL-Qura University, Mecca 24382, Saudi Arabia; aomehder@uqu.edu.sa; 10 Department of Food Industries, Faculty of Agriculture, Damietta University, Damietta 34511, Egypt; emanmail2005@yahoo.com

**Keywords:** secreted phospholipase A_2_, chronic inflammatory diseases, proinflammatory mediators, anti-inflammatory drugs, sinapic acid, hemorrhagic activity

## Abstract

Human Group IIA secreted phospholipase A_2_ (sPLA_2_-IIA) enzyme plays a crucial role in several chronic inflammatory diseases such asasthma, atherosclerosis, gout, bronchitis, etc. Several studies showed that the antioxidants exert an anti-inflammatory function by inhibiting the sPLA_2_-IIA enzyme. Hence, the present study evaluated an antioxidant molecule, sinapic acid, for sPLA_2_-IIA inhibition as an anti-inflammatory function. Initially, the antioxidant efficacy of sinapic acid was evaluated, and it showed greater antioxidant potency. Further, sinapic acid inhibited 94.4 ± 4.83% of sPLA_2_-IIA activity with an IC_50_ value of 4.16 ± 0.13 µM. The mode of sPLA_2_-IIA inhibition was examined by increasing the substrate concentration from 30 to 120nM and the calcium concentration from 2.5 to 15 mM, which did not change the level of inhibition. Further, sinapic acid altered the intrinsic fluorescence and distorted the far UltraViolet Circular Dichroism (UV-CD) spectra of the sPLA_2_-IIA, indicating the direct enzyme-inhibitor interaction. Sinapic acid reduced the sPLA_2_-IIA mediated hemolytic activity from 94 ± 2.19% to 12.35 ± 2.57% and mouse paw edema from 171.75 ± 2.2% to 114.8 ± 1.98%, demonstrating the anti-inflammatory efficiency of sinapic acid by in situ and in vivo methods, respectively. Finally, sinapic acid reduced the hemorrhagic effect of *Vipera russelli* venom hemorrhagic complex-I (VR-HC-I) as an anti-hemorrhagic function. Thus, the above experimental results revealed the sinapic acid potency to be an antioxidant, anti-inflammatory and anti-hemorrhagic molecule, and therefore, it appears to be a promising therapeutic agent.

## 1. Introduction

Inflammation is a defensive process and a necessary prerequisite to healing the tissue injury that occurs due to physical, chemical, or biological agents. However, if the inflammation remains beyond its defensive role, it leads to serious consequences such as systemic shock, circulatory collapse, and local tissue injury [1]. Studies have shown that secreted phospholipase A_2_ group IIA (sPLA_2_-IIA) enzymes play a significant role in oxidative stress [2] and inflammatory diseases [3,4]. In healthy people, the concentration of sPLA_2_-IIA is minimal (3 ng/mL) but increases significantly (250–500 ng/mL) during infection and injuries [5]. The sPLA_2_-IIA concentration has been elevated in most inflammatory fluids of patients with rheumatoid arthritis [6], asthma [7], atherosclerosis [8], and acute respiratory distress syndrome [9], as well as a biomarker for cardiovascular complications [10,11], sepsis [12] and transplant rejection [13].

**Figure 1:**
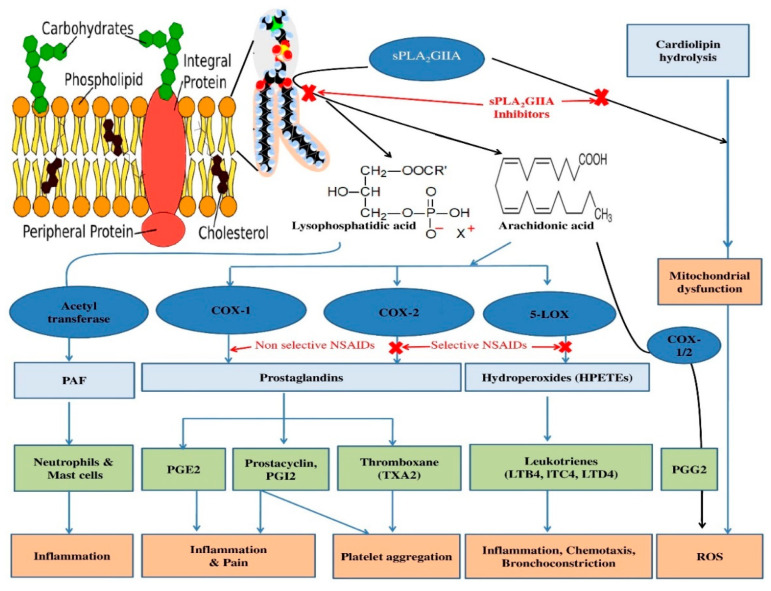
Schematic representation of sPLA_2_-IIA mediated inflammatory pathways, ROS production and check points of anti-inflammatory molecule/drugs.

The sPLA_2_-IIA enzyme catalyzes membrane phospholipid into arachidonic acid and lysophosphatidic acid. Arachidonic acid is converted into inflammatory mediators such as thromboxane, leukotriene, prostaglandins, and prostacyclins. Lysophosphatidic acid is catalyzed to a platelet activating factor (PAF) that further intensifies the inflammatory condition (Figure 1). Furthermore, the arachidonic acid pathway produces loads of reactive oxygen species (ROS), which contribute to the defensive function by destroying inflowing pathogens [14,15]. However, the persistence of ROS after the defensive role causes deleterious complications [16]. Furthermore, they play an important role in several inflammatory diseases such as ARDS, COPD, chronic bronchitis, asthma [17], rheumatoid arthritis [18], and Alzheimer’s disease [19].

The arachidonic acid pathway-mediated ROS production modulates the cPLA_2_ and iPLA_2_ functions that enhance the production of arachidonic acid and free radicals [20]. Interestingly, the ROS increases the sPLA_2_-IIA activity and lipid peroxidation that modulate the downstream reactions, which further increase the proinflammatory mediators. Therefore, a single bioactive molecule with both sPLA_2_-IIA inhibitory and antioxidant activities may become a more effective anti-inflammatory agent. Till today, Non-Steroidal Anti-Inflammatory Drugs (NSAIDs) are widely used to control chronic inflammatory disorders [21,22]. NSAIDs limit the COX-1/2 enzymes but have no effect on the generation of leukotrienes and PAF [23]; they continue to cause inflammation (Figure 1). Furthermore, the prolonged use of NSAIDs leads to several complications such as hepatotoxicity, renal injury, hypertension, cardiovascular risks, and gastrointestinal toxicity [24,25,26,27]. The specific sPLA_2_-IIA inhibitors such as varespladib (LY315920) and varespladib-methyl (LY333013) were examined in clinical trials, where they were used to treat patients with cardiovascular complications [28,29], but they failed to demonstrate the therapeutic effects. Drugs such asLY315920NA, ginkgetin and petrosaspongiolide M were not successful even though they limit the sPLA_2_-IIA activity at nanomolar concentrations. The unsuccessfulness of these sPLA_2_-IIA inhibitors may be due to the problem associated with formulation or their cytotoxic nature [30,31]. As a result, there is an urgent need for safe and effective sPLA_2_-IIA inhibitors from natural resources with minimal or no adverse effects [32].

Antioxidants such as flavonoids, phenols, and retinoids scavenge ROS and prevent lipid peroxidation, and they further limit the sPLA_2_-IIA-mediated arachidonic acid cascade [14]. In our initial study on pharmaceutically important bioactive molecules, sinapic acid, an antioxidant found in dietary sources [33], has been shown to interfere with the pathways connected to inflammation. Sinapic acid plays a protective role against oxidative stress disorders, as shown in [34], and another study has shown the anti-inflammatory effect by down regulating the synthesis of iNOS and COX-2 in murine macrophage cell lines [35]. Sinapic acid is also documented for its anti-inflammatory effects by inhibiting IL-1β [36], NF-κB [35],reducing the risk of inflammatory colitis in mice by suppressing malondialdehyde, TNF-α and myeloperoxidase expression [37],and reducing carrageenan-induced edema [35]. Therefore, we hypothesized our research to evaluate thepotency of sinapic acid for neutralizing the PLA_2_-IIA enzyme and its inflammation responses.

## 2. Materials and Methods

### 2.1. Materials

Sinapic acid, gallic acid (GA), thiobarbituric acid (TBA), Sephadex (G25, G50, and G75), CM-Sephadex-25, 2, 2-diphenyl-1-picrylhydrazyl radical (DPPH), Ultima Gold Scintillation Cocktail, and dimethyl sulfoxide (DMSO) areproducts of Sigma-Aldrich, St. Louis, MO, USA. ^14^C-oleic acid was procured from Perkin Elmer Life Sciences Inc. in Boston, MA, USA. The venom of *Viper russelli* was purchased from Irula Cooperative Society Ltd., Tamil Nadu, India. All reagents and chemicals used in the investigation were of superior quality.

### 2.2. Animals

Swiss albino mice (weighing around 20–25 g, males) were procured from the University Animal House Facility (AHF), Mangalore University, Mangalore, India. Animals were maintained and handled according to the guidelines of the Indian National Regulations for Animal Research. In the present study, we conducted the experiments according to the guidelines of Mangalore University’s Institutional Animal Ethical Committee (No: MU/AZ/504(a)/IAEC/2015-2016).

### 2.3. Human Biological Fluid

Institutional Human Ethical Committee (IHEC), Mangalore University, Mangalore, India, permitted the usage of human blood samples (IHEC-No. MU/IHEC/2018/7). The blood samples were collected from volunteers after obtaining the consent letter.

### 2.4. Purification of sPLA_2_-IIA

The sPLA_2_-IIA was purified from *Viper russelli* venom as per the protocol of Kasturi and Gowda [38]. The purity of sPLA_2_-IIA was tested by sodium dodecyl-sulfate polyacrylamide gel electrophoresis [39]. The sPLA_2_-IIA of *Vipera russelli* venom was generally used to study the mode of action of human inflammatory sPLA_2_-IIA because of the simple purification procedure, availability, close structural similarities, and catalytic action compared to human sPLA_2_ IIA [40].

The human and snake venom sPLA_2_ enzymes share similar functional and biological properties such as edema, pain, muscle injury and leukocyte influx [41]. It was also reported that the binding pattern of a known inhibitor with human and venom phospholipase A_2_ was very similar [42]. Hence, it is suggested the use of snake venom PLA_2_ as a tool for investigating a new pharmacological inhibitor of human sPLA_2_-IIA [42].

### 2.5. Molecular Docking

The structures of phospholipase A_2_ (PLA_2_) were downloaded from the Protein Data Bank (PDB ID: 1POE and 3H1X). Structures of sinapic acid and genistein were drawn and analyzed with ChemDraw Ultra 12.0. The three-dimensional coordination was derived through the PRODRG online server [43]. The potential active pockets for PLA_2_ protein were determined and identified from both of CASTp server and reference [44]. During the process, intermediary steps, such as grid box creation, energy optimization, protein and ligand preparations, were established through the Graphical User Interface program of AutoDock Tools (ADT). AutoDock prepared the data and saved the prepared file in the required PDBQT format. Using the available information about chosen protein and ligand, AutoDock Vina was used for the docking process along with grid box characteristics in the configuration file. AutoDock Vina employs an iterated local search global optimizer to process the submitted data [45]. During the docking procedure, the option was selected to consider both the ligand and protein as rigid. Following the completion of the scheduled docking runs, the variable conformations of the protein with ligands were obtained as binding methods with their respective binding affinity. The stable confirmation mode with the optimum best interaction was selected and was the one that represented the lowest binding affinity; the same was picked and aligned alongside the receptor structure for further investigation [46].

### 2.6. Estimation of Antioxidant Activity

The antioxidant activity of sinapic acid was estimated by DPPH radical scavenging activity, as described by Blois [47], anti-lipid peroxidation activity, as described by Gutteridge [48], and reducing power activity, as described by Oyaizu [49]. The antioxidant activity was expressed as percent radical scavenging activity.

### 2.7. Secreted Phospholipase A_2_ Assay (sPLA_2_-IIA)

Autoclaved *E. coli* cells labeled with ^14^C-oleic acid were used as a substrate for estimation of sPLA_2_-IIA activity [50,51]. Briefly, the reaction mixture (350 µL) consists of 3.18 × 10^9^ autoclaved *E. coli* cells, calcium (5 mM), Tris-HCl buffer (100 mM), enzyme, and water. The 30 µL *E-coli* substrate was added and incubated at 37 °C for 60 min. In total, 2N HCl (100 µL) and 100 μL fatty acid-free BSA (10%) were added, vortexed and centrifuged at 20,000× *g* for 5 min. A total of 140 µL supernatant containing ^14^C-oleic acid was collected with caution and was added to a scintillation cocktail, and the ^14^C radioactivity was measured.

### 2.8. Inhibition of sPLA_2_-IIA Activity

Sinapic acid was dissolved in a small amount of DMSO and made up to the appropriate concentration with Tris-HCl buffer. sPLA_2_-IIA inhibition was performed by taking different concentrations of sinapic acid. Genistein was used as the standard molecule as it was a proven sPLA_2_-IIA inhibitor and an anti-inflammatory molecule [52]. The maximum concentration of DMSO used in the experiment was 0.022%. The Graph pad Prism version 5.0 (GraphPad Software, San Diego, CA, USA) software was employed to calculate the IC_50_ value.

### 2.9. The Effect of Concentrations of Substrate and Calcium on sPLA_2_-IIA Inhibition

The assay was carried out with and without IC_50_ concentration of sinapic acid as described above. The effect of substrate concentration on sPLA_2_-IIA inhibition was studied by increasing its concentration from 30 to 120 nmoles. The effect of calcium concentrations on sPLA_2_-IIA inhibition was examined by increasing its concentration from 2.5 to 15 mM.

### 2.10. Intrinsic Fluorescence Study

The fluorescence intensity of the sPLA_2_-IIA enzyme was measured with and without sinapic acid using the Horiba Jobin Yvon Fluorolog-3 spectrofluorometer. The standard reaction mixture (2.0 mL) in a 1 cm path length cuvette consists of sPLA_2_-IIA (20 µg/mL) and sinapic acid concentrations ranging from 0.02 to 0.10 µM. The spectra of the fluorescence were measured between the wavelength of 300 and 380 nm. The tryptophan standard was employed to correct the measurements empirically [53].

### 2.11. The Far UV-Circular Dichroism Study

The UV-CD spectra of sPLA_2_-IIA (30 µg/mL) were recorded with/without sinapic acid in a reaction mixture using a Jasco J-810 spectropolarimeter. The quartz cuvette was used to record the spectra of sPLA_2_-IIA between 200 and 240 nm at room temperature. The bandwidth was 1 nm, and the response time was set for 2 s. Ten scans in total were carried out to obtainthe final spectrum. The spectrum of the blank solution containing the standard reaction mixture was subtracted to correct the protein spectra. The secondary structure of sPLA_2_-IIA was calculated using the K2D3 software (http://cbdm-01.zdv.uni-mainz.de/~andrade/k2d3/).

### 2.12. Study of Reversibility of sPLA_2_-IIA Inhibition

The sPLA_2_-IIA with IC_50_ concentration of sinapic acid in 350 µL standard reaction mixture was preincubated and then subjected to dialysis (MW cut off of bag is 3000–6000) for twenty-four hours by changing two buffers. The sPLA_2_-IIA activity was determined before and following the dialysis procedure.

### 2.13. Neutralization of Indirect Haemolytic Activity

The experiment was conducted as per the method of Boman and Kaletta [54]. The human RBC (1 mL) and egg yolk (1 mL) in 8 mL of PBS were mixed fresh as a substrate for indirect hemolytic activity. The inhibitor (sinapic acid) was preincubated with sPLA_2_-IIA (30 µg) at 37 °C for 30 min, and 1 mL of a substrate was added and allowed for the reaction for 45 min at 37 °C. In total, 9 mL of ice-cold PBS was added to halt the reaction. The suspension was vortexed and centrifuged for 20 min at 1500× *g*. The hemolytic activity in terms of released hemoglobin was measured at 530 nm. The sPLA_2_-IIA enzyme without sinapic acid was the positive control.

### 2.14. Neutralization of Edema Inducing Activity of sPLA_2_-IIA

The assay was performed as per the method of Yamakawa et al. [55], slightly modified by Vishwanath et al. [56]. The sPLA_2_-IIA (5 µg) with different concentrations of sinapic acid, making up a total of 20 µL, was injected into the plantar surface of the right hind footpad of mice (weighing 20–25 g). The saline was injected to the respective left hind limb as negative controls. The animals were euthanized after 45 min by administering anesthesia (30 mg/kg of pentobarbitone i.p.), and hind limbs were amputated at the ankle joint and were weighed individually. The edema percentage was calculated using the following formula

$$Edema\;ratio\;=\;\frac{Weight\;of\;the\;edematous\;leg}{Weight\;of\;normal\;leg\;\left(saline\;injected\right)}\;\times \;100$$

### 2.15. Hemorrhagic Activity of sPLA_2_-IIA

The hemorrhagic activity of sPLA_2_-IIA was estimated as described by Kondo H and Venkatesh M [57,58]. Briefly, 10 μg of the hemorrhagic complex containing 5:2 ratio of sPLA_2_-IIA enzyme and nonenzymatic peptide (Vipera neurotoxin-II, VNTx-II) was injected subcutaneously (s.c.). The mice were euthanized after three hours, the skin was removed, and hemorrhagic spots were measured on the dorsal surface. Saline alone was injected as the control. The preincubated hemorrhagic complex was injected with different concentrations of sinapic acid for the inhibition studies.

### 2.16. Statistical Analysis

The test results were given as the mean standard deviation of three determinations. Graph Pad Prism Version 5.0 was used to calculate IC_50_ values. Percent inhibition was calculated from the difference between the control receiving vehicle and the inhibitor-treated animals.

## 3. Results

### 3.1. Molecular Docking

**Figure 2:**
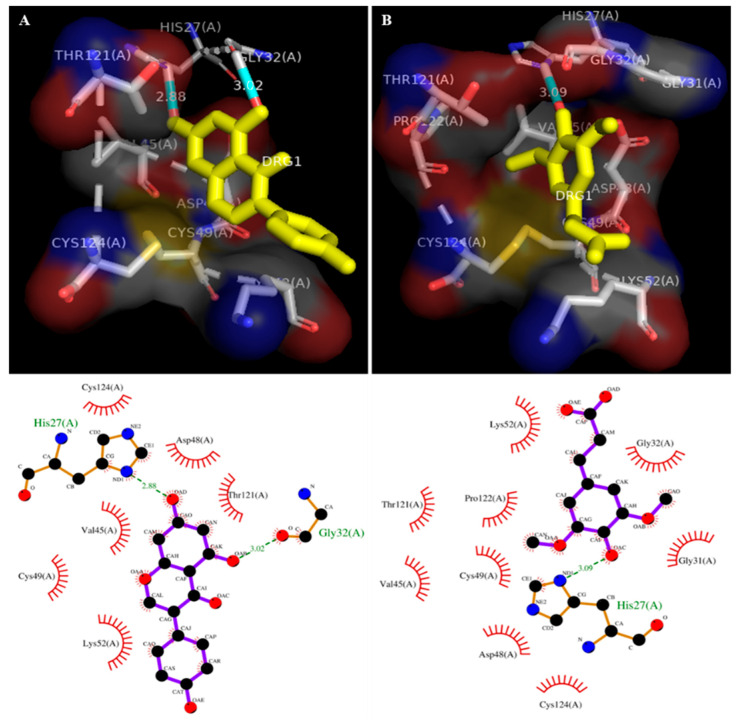
Docked images of sPLA_2_-IIA (1POE) with genistein (standard) and sinapic acid: 2D Ligplot analysis and the crystal structure of sPLA2-IIA (1POE) with standard genistein (**A**) and sinapic acid (**B**). Hydrogen bonds are presented by the green dashed line, and the unit of distance was set as Å.

The molecular docking study was carried out to analyze the enzyme–inhibitor interaction. The sinapic acid interacted with the human sPLA_2_-IIA (1POE) enzyme, and it showed the binding energy of −7.6 (E-value). The sinapic acid interacted with the active site conserved amino acid Asp48 through hydrogen bonding and hydrophobic interaction with Cys124, Val45, Cys49, Thr121, Pro122, Lys52, Gly32, and Gly31 (Figure 2B and Table 1). Similarly, the binding energy of standard genistein was −7.2, which interacted with active site Asp48 and Lys52 through hydrogen bonding and showed hydrophobic interaction with Val45, Cys49, Thr121, and Cys124 residues (Figure 2A and Table 1).

### 3.2. Estimation of Antioxidant Activities

The antioxidant potency of sinapic acid was examined by the DPPH method, reducing power assay and anti-lipid peroxidation activity. The sinapic acid scavenged the DPPH radicals to 89% ± 2.13, whereas the ascorbic acid (standard) showed 96% ± 1.78. The reducing power of sinapic acid was 63.5% ± 2.05, compared to quercetin (standard), which showed 66.5% ± 1.82. The anti-lipid peroxidation was 80.6% ± 1.07, whereas α-lipoic acid (standard) exhibited 89% ± 1.69 (Table 2).

The antioxidant activity of sinapic acid was evaluated by DPPH radical scavenging, reducing power assay and anti-lipid peroxidation.

### 3.3. Inhibition of sPLA_2_-IIA

**Figure 3:**
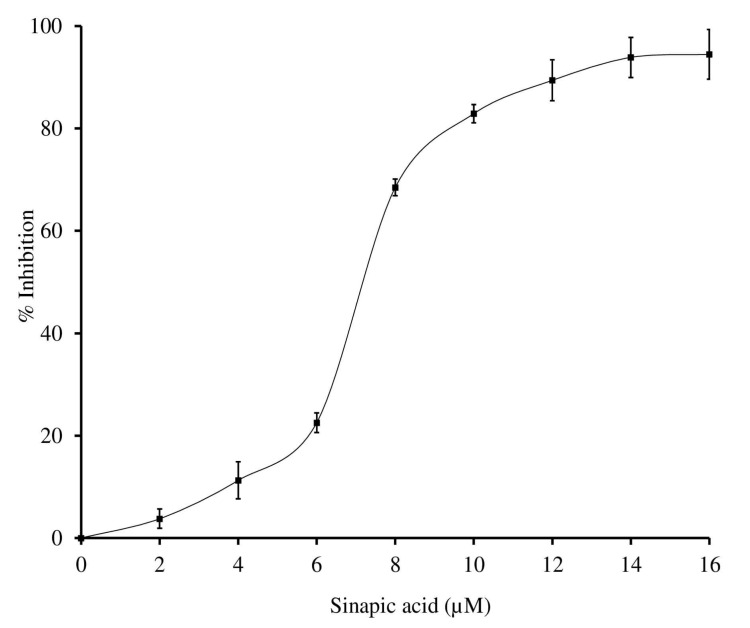
Inhibition of sPLA2-IIA enzyme by sinapic acid: sPLA2-IIA enzyme was incubated with sinapic acid (2–16 µM) at 37 °C for 60 min. Enzyme activity was measured by measuring the radiation of ^14^C by liquid scintillation spectrometer. Enzyme inhibition was noted as a percentage of control. The data represents mean ± SD (n = 3).

Further, the sinapic acid was employed to inhibit the inflammatory sPLA_2_-IIA enzyme. It potentially inhibited the sPLA_2_-IIA enzyme to the extent of 94.4% ± 4.83 at 16 µM concentration with an F-statistic value of 0.0031 and *p*-value of 0.9969 (Figure 3). The IC_50_ value of sinapic acid was calculated by the software Graphpad Prism 5.0, and it was shown to be 4.16 ± 0.13 µM, whereas the standard genistein was 11.75 µM (historical IC_50_ value) (Table 3) [52].

### 3.4. Effect of Calcium and Substrate Concentration on sPLA_2_ IIA Inhibition

**Figure 4:**
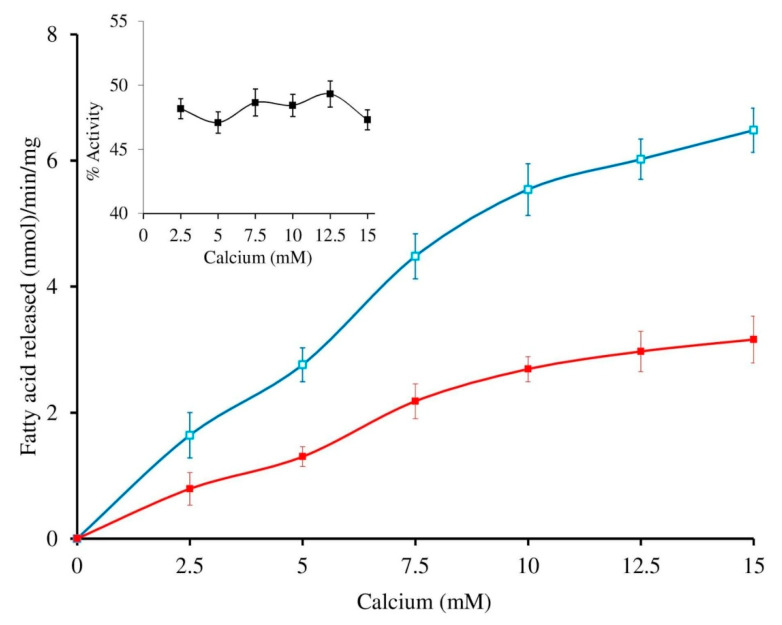
Effect of calcium concentration on sPLA2-IIA inhibition: The sPLA2 IIA activity was measured with calcium concentration ranges from 2.5 to 15 mM with (■) and without (□) IC_50_ concentration of sinapic acid. The sPLA_2_-IIA inhibition is shown in the inlet. The data are expressed as mean ± standard deviation (n = 3).

**Figure 5:**
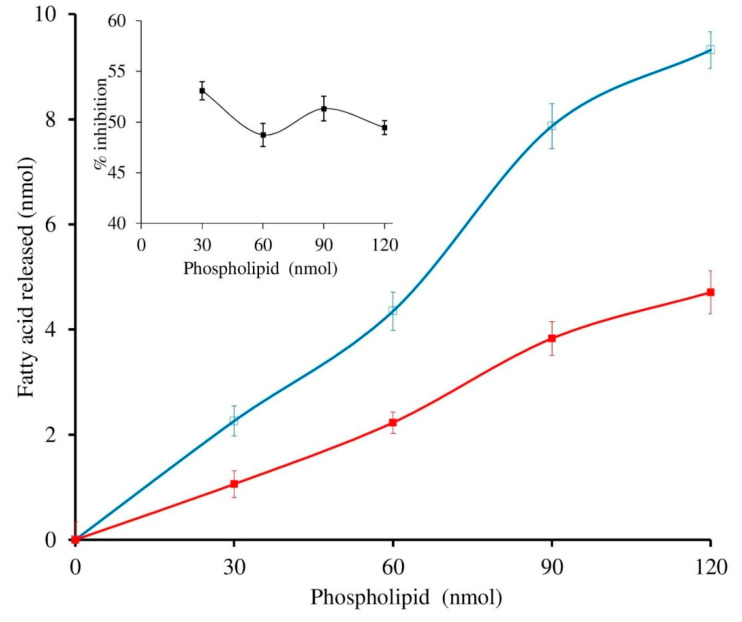
Effect of substrate concentration on sPLA2-IIA inhibition: The sPLA2 IIA activity was measured with the substrate concentrations ranging from 30 to 120 μL with (■) and without (□) IC_50_ concentration of sinapic acid. The sPLA_2_-IIA inhibition is shown in the inlet. The data are expressed as mean ± standard deviation (n = 3).

The sPLA_2_-IIA activity was measured with and without sinapic acid (IC_50_ concentration) by increasing the calcium concentration from 2.5 to 15 mM, the activity of the enzyme was increased linearly and maintained the constant inhibition of 49.34% ± 1.35 over all the ranges of the calcium concentrations (Figure 4). Furthermore, sPLA_2_-IIA activity was measured with and without IC_50_ concentration of sinapic acid by increasing substrate concentration from 30 to 120 nmoles; the enzyme activity increased linearly and maintained the constant inhibition of 48.43% ± 1.76 over all the ranges of substrate concentrations (Figure 5).

**Figure 6:**
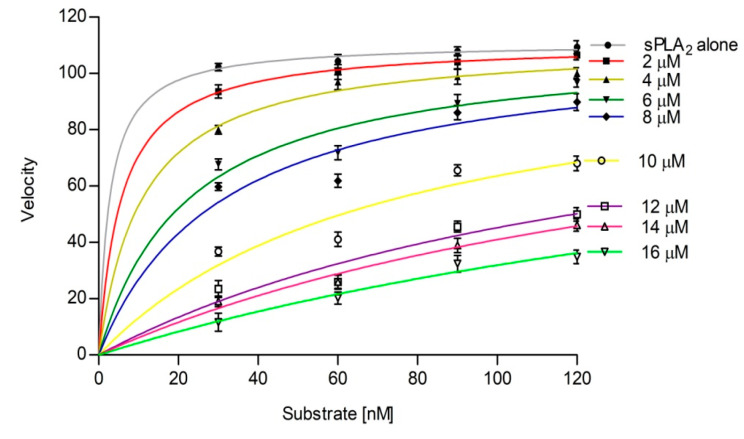
Inhibition constant (Ki) of sinapic acid for sPLA2-IIA. The primary data fit the competitive inhibition model, and Ki was determined byincreasing the concentrations of sinapic acid by using Graph pad prism 5.0. Values plotted mean ± standard deviation (n = 3).

The inhibition constant (Ki) was determined by fitting the data to the competitive inhibition model in GraphPad Prism 5.0 via nonlinear regression analysis of competitive enzyme kinetics [59] (Figure 6). The Ki of sinapic acid for sPLA_2_-IIA inhibition was found to be 2.711 ± 1.19. The Ki and IC_50_ values are often used to compare the relative potency of inhibitors. Smaller Ki values denote tight binding, and if the Ki value is less than the IC_50_ value, it indicates competitive inhibition [60].

### 3.5. Intrinsic Fluorescence Study

**Figure 7:**
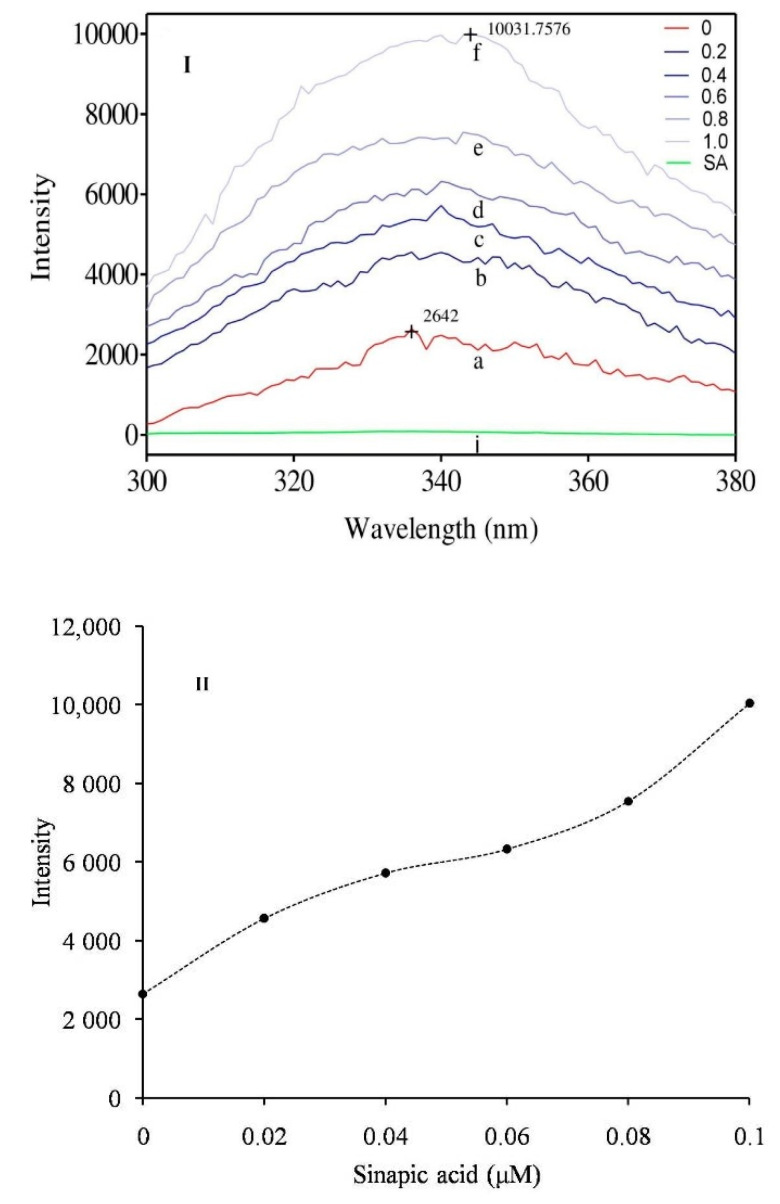
Intrinsic fluorescence spectra of sPLA2-IIA enzyme: (**I**) Fluorescence spectra of sPLA2-IIA enzyme (20 µg/mL) alone (a), with sinapic acid concentrations of 0.02 μM (b), 0.04 μM (c), 0.06 μM (d), 0.08 μM (e) and 0.1 μM (f). (**II**) The graph showed maximum fluorescence intensity of sPLA_2_-IIA enzyme of each concentration of sinapic acid.

The altered intrinsic fluorescence spectrum of the enzyme indicates the structural changes due to interaction with the inhibitor. Sinapic acid altered the relative intrinsic fluorescence of the sPLA_2_-IIA enzyme in accordance with the inhibitor concentration (0.02 to 0.1 μM). The maximum fluorescence intensity of sPLA_2_-IIA was noted at 338 nm and shifted to the higher wavelength of 344 nm in the presence of sinapic acid at 0.1 μM concentration (Figure 7I,II).

### 3.6. Circular Dichroism (CD) Study

**Figure 8:**
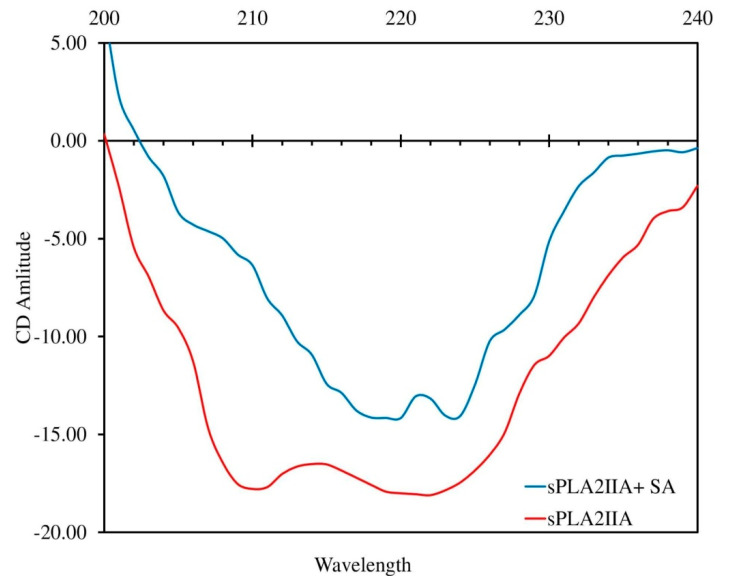
Circular Dichroism spectra of sPLA2-IIA with and without sinapic acid: The far-UV CD spectra of sPLA_2_-IIA alone (red line) and with sinapic acid (IC_50_ concentration) (blue line) were recorded between 200 and 240 nm using a Jasco J-810 spectropolarimeter.

The change in the secondary structure of the enzyme implies the direct interaction with the inhibitor. The CD spectrum of sPLA_2_-IIA with and without IC_50_ concentration of sinapic acid was recorded, which exhibited two major negative bands at 210 and 222 nm (red line). In the presence of sinapic acid (IC_50_ concentration), the negative bands were significantly reduced and abruptly shifted to longer wavelengths of 220 and 224 nm, respectively (Figure 8). The sPLA_2_-IIA spectra were corrected by subtracting spectra of the blank solution containing 100 mM Tris-HCl buffer (pH 7.4) and 5 mM calcium. The K2D3 software was used to determine the secondary structure of the sPLA_2_-IIA enzyme (Table 4).

### 3.7. Determination of Binding Characteristics

The reversibility of sPLA_2_-IIA inhibition was studied by subjecting the preincubated reaction mixture to dialysis. The sPLA_2_-IIA activity was measured before and after the dialysis. The percentage of sPLA_2_-IIA inhibition before and after the dialysis was found to be 50.2% ± 2.3 and 47.8% ± 1.55, respectively.

### 3.8. Neutralization of Indirect Haemolytic Activity

**Figure 9:**
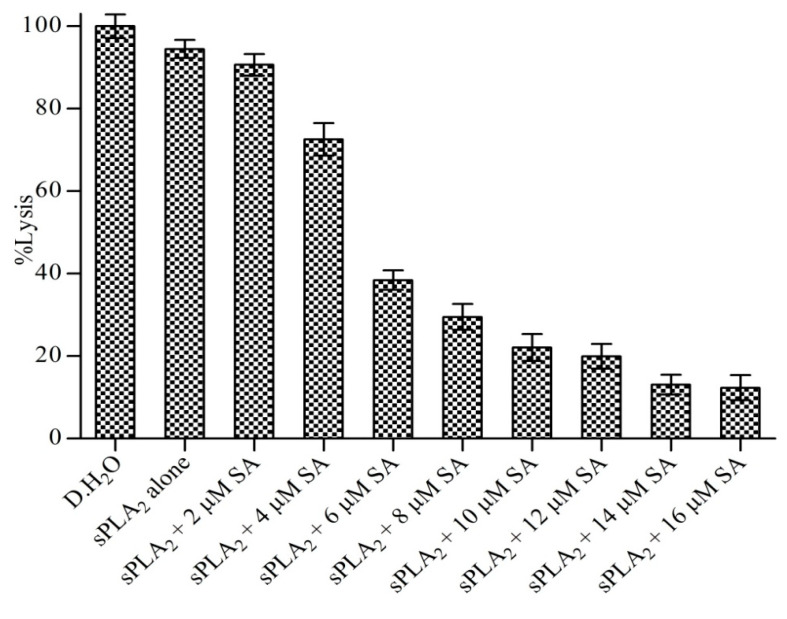
Neutralization of sPLA2-IIA induced indirect hemolytic activity: The reaction was initiated by adding 1 mL of substrate to preincubated sPLA_2_-IIA with indicated concentration sinapic acid and incubated at 37 °C for 30 min. The released hemoglobin was measured by reading the optical density at 540 nm. Data represent the mean standard deviation (n = 3).

Sinapic acid was subjected to neutralizing indirect hemolytic activity of the sPLA_2_-IIA enzyme. Sinapic acid reduced the indirect hemolytic activity of sPLA_2_-IIA in a concentration-dependent manner. The sPLA_2_-IIA (30 μg) alone causes erythrocyte lysis to 94% ± 2.19, which was reduced to 12.35% ± 2.57 by sinapic acid at a concentration of 16 μM (Figure 9). Distilled water served as a positive control (100% lysis).

### 3.9. Neutralization of sPLA_2_ IIA Induced Mouse Paw Edema

**Figure 10:**
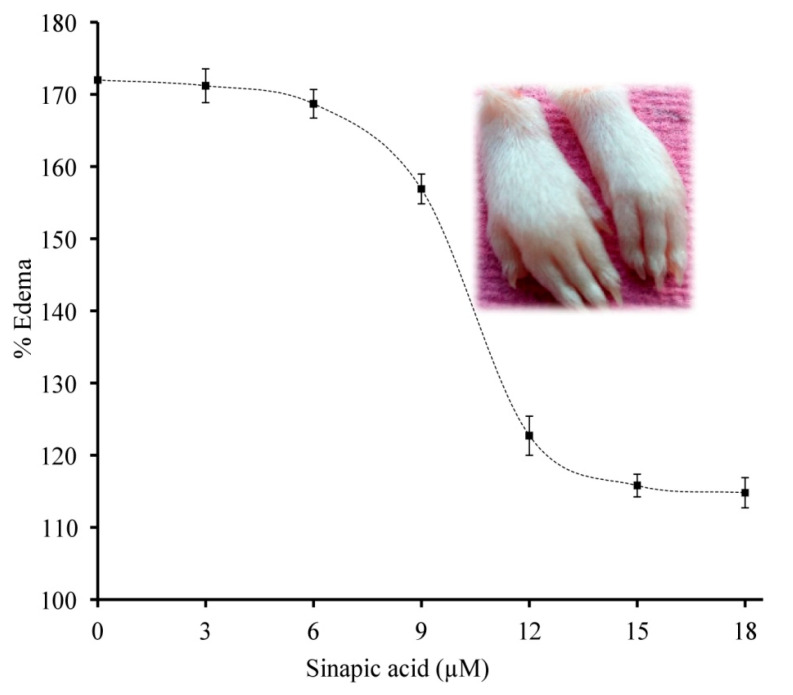
Neutralization of sPLA2-IIA induced edema: The preincubateds PLA_2_-IIA enzyme (5 µg) with the indicated concentrations of sinapic acid injected to the right hind footpad of mice (20 µL). The mice were euthanized after 45 min and legs were amputated at the ankle, and the edema ratio was calculated. The data are shown in mean ± standard deviation (n = 3).

The different doses of sinapic acid (3–18 μM) were preincubated with sPLA_2_-IIA and injected into the right hind paw of mice, and saline injected to the left hind paw served as the control. Sinapic acid reduced the edema from 171.75% ± 2.2 to 114.8% ± 1.98 at 18 μM concentration, and the reduced percentage of sPLA_2_-IIA-induced edema was 79.12% ± 1.52 (Figure 10).

### 3.10. Neutralization of Haemorrhagic Activity

**Figure 11:**
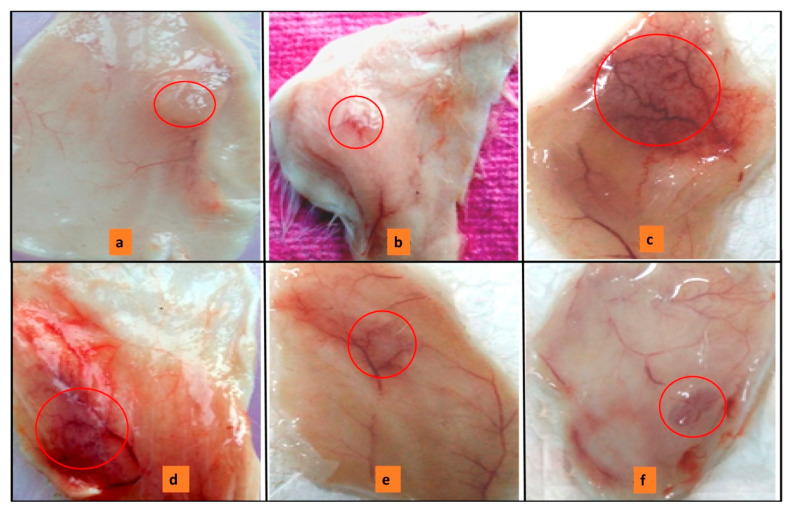
ReducingVR-HC-I mediated hemorrhagic activity by sinapic acid:Mice injected with 10 µg of sPLA_2_-IIA (**a**), 10 µg of VNTx-II alone (**b**), 10 µg of VR-HC-I (**c**). The injection of preincubated 10 µg of VR-HC-I with sinapic acid of 5 µM (**d**), 10 µM (**e**), 15 µM (**f**). The mice were sacrificed after 3 h, and hemorrhagic spots were recorded using the graph sheet.

This study reveals the synergistic effect of sPLA_2_-IIA and nonenzymatic peptides. The *Vipera russellii* sPLA_2_-IIA and *Vipera russellii* neurotoxic nonenzymatic peptide (VNTx-II) in the 5:2 molar ratio is called *V. russelli* Hemorrhagic Complex-I (VR-HC-I) [58] and was administered to mice intradermally. VR-HC-I induced the hemorrhage at the injection site (Figure 11c). Neither sPLA_2_-IIA nor VNTx-II independently showed the hemorrhagic effect (Figure 11a,b respectively). The mice were injected with preincubated VR-HC-I with sinapic acid (5, 10 and 15 μM), which reduced the hemorrhagic potential (Figure 11d–f respectively). Sinapic acid significantly neutralized the hemorrhagic activity at 15 μM concentration.

## 4. Discussion

Sinapic acid is rich in fruits such as orange, mango, avocado, strawberries, and raspberries [61,62,63], vegetables such as garlic, onions, cabbage [64,65], and legumes such as horse grams [66]. Among them, avocado, garlic, and horse gram are well documented for their anti-inflammatory activity [67,68]. The sinapic acid reported no cytotoxic effect on V79 cells [69], and there was no effect on lactate dehydrogenase activity and serum creatine kinase in broilers, suggesting that there are no effects on the brain, liver, kidneys, and cardiac muscle [70]. Thus, sinapic acid from the food items was demonstrated as a non-toxic and therapeutically important molecule.

The in silico molecular docking study is important at the early stage of drug discovery because it provides basic knowledge of binding energy, pattern and binding affinity. The docking results of sinapic acid with sPLA_2_-IIA (1POE) exhibited greater binding energy (E value −7.6), which was slightly higher than the binding energy of standard genistein (E value −7.2). Most of the sPLA_2_-IIA inhibitors interfere with the catalytic site by binding to His 47/Asp 48 and decreases the catalytic activity by weakening Ca^2+^ interaction [71,72]. The sinapic acid interacted with the active site amino acid Asp48 through hydrogen bonding and showed hydrophobic interaction with few amino acids. Therefore, sinapic acid was assumed to be a potent inhibitor of sPLA_2_- IIA enzyme (Table 1).

The reactive oxygen species (ROS) and their role in human disease has become an important aspect of disease management. The sPLA_2_-IIA-mediated ROS generation through activation of NADPH oxidases [73] is an important pathway as it is known to be involved in the activation of cPLA_2_ and ERK1/2 [74], leading to the release of arachidonic acid. Furthermore, the hydroxyl radicals that formed during inflammation attack membrane glycerophospholipids and initiate lipid peroxidation [73]. Hence, sinapic acid was evaluated for its antioxidant efficacy. Sinapic acid effectively scavenged the DPPH radical, reduced the ferric ions, and demonstrated its ability to protect lipid peroxidation. Thus, it was concluded that sinapic acid, if developed as an anti-inflammatory drug, limits free radicals and their intermediates released during inflammatory pathologies.

The results illustrated that the sinapic acid exhibited greater binding energy (docking study) and better antioxidant potency and, hence, was further examined for its anti-inflammatory activity. Sinapic acid potentially inhibited the sPLA_2_-IIA enzyme in a concentration-dependent manner and with a comparatively low IC_50_ value. The genistein was taken as a standard molecule in this study as it is a well-known anti-inflammatory and antioxidant molecule [75,76,77]. The kinetics study of sPLA_2_-IIAinhibition was performed using Graph pad prism software 5.0, which suggested that the sinapic acid is a competitive inhibitor of sPLA_2_-IIA.

Many sPLA_2_-IIA inhibitors limit the enzyme activity either by chelating calcium (metal ion) or binding to substrates. Inhibitors such as lipocortin I and II bind sPLA_2_-IIA enzymes non-specifically and affect the quality of the lipid interface [78]. Therefore, we examined the effect of substrate and calcium concentrations on sPLA_2_-IIA inhibition. The findings exhibit that the inhibition of sPLA_2_-IIA by sinapic acid was not dependent on calcium or substrate concentrations.

Many sPLA_2_-IIA inhibitors were reported to alter fluorescence spectra [79]. The structural change in the enzyme upon interaction with the inhibitor alters the intrinsic fluorescence. The aromatic amino acids of protein such as tryptophan, tyrosine, and phenylalanine are responsible for intrinsic fluorescence. The quantum yield, intensity, and wavelength of maximum fluorescence emission depend upon the microenvironment of these aromatic amino acids. The sinapic acid shifts the maximum fluorescence spectrum of sPLA_2_-IIA towards the shorter wavelength and increases the fluorescence intensity as the polarity of the solvent surrounding the aromatic amino acids decreases [80,81]. Sinapic acid alone does not show any fluorescence, indicating that sinapic acid interacts directly with the sPLA_2_-IIA enzyme.

To substantiate fluorimetry results, a circular dichroism (CD) study was carried out. The studies showed that significant changes occur in the secondary structure of sPLA_2_-IIA upon inhibitors binding [82]. In the present study, the sinapic acid interaction with the sPLA_2_-IIA enzyme caused significant changes in the secondary structure (Figure 8). Hence, it is indisputably concluded that sinapic acid inhibits sPLA_2_-IIA by irreversibly binding to the active site.

The reversibility of sPLA_2_-IIA inhibition was examined by measuring the percentage of inhibition before and after the dialysis of the reaction mixture. The inhibition percentages before and after the dialysis were almost the same. Hence, it again is implicit that sinapic acid irreversibly binds to the sPLA_2_-IIA enzyme.

The indirect hemolytic assay is an indirect way of estimating sPLA_2_-IIA activity using egg yolk phospholipid and washed erythrocytes as substrates [83]. Sinapic acid efficiently neutralized the sPLA_2_-IIA-mediated hemolysis in a dose-dependent way. Thus, sinapic acid neutralizes sPLA_2_-IIA enzyme activity irrespective of the nature of the substrate because the sinapic acid binds to the enzyme irreversibly.

It has been observed that the in vitro experiments show positive results but fail to show efficiency in the in vivo studies. This could be due to the heterogeneity of the environment in the in vivo models. Animal experiments are important for researchers as they provide peer knowledge of pharmacodynamics and pharmacokinetics in the early stages of drug discovery [84]. Therefore, the effectiveness of the sinapic acid in neutralizing the sPLA_2_-IIA-induced inflammatory response in the Swiss albino mice was evaluated. Sinapic acid reduced inflammatory edema to a greater extent. Thus, sinapic acid demonstrated the in vivo efficacy by neutralizing the sPLA_2_-IIA mediated inflammatory response.

In the living system, protein–protein interactions lead to pharmacological damage due to the synergistic effect [85]. For example, the interaction of human sPLA_2_-IIA and vimentin (an intracellular protein) further exacerbates inflammatory pathologies. Interestingly, the addition of LY311727 (sPLA_2_-IIA inhibitor) causes substantial structural displacement in the amino terminus of the sPLA_2_-IIA enzyme, and that is sufficient to minimize its interaction with the vimentin. The interaction between sPLA_2_-IIA and nonenzymatic peptides is synergetic in snake bites and leads to increased hemorrhage [58]. In the present study, sinapic acid significantly reduced the synergistic hemorrhagic effect of *V. russelli* Haemorrhagic Complex-I (sPLA_2_-IIA and *V. russelli* neurotoxic nonenzymatic peptide) (Figure 10).

## 5. Conclusions

Activated sPLA_2_-IIA generates proinflammatory lipid mediators and oxygen-free radicals that intensify the status of oxidative stress disorders and chronic inflammatory diseases. The present study evaluated the sinapic acid from a dietary source for both antioxidant potency and sPLA_2_-IIA inhibition as an anti-inflammatory function, and the result showed that sinapic acid exhibit both the potencies to a better extent. Further, sPLA_2_-IIA inhibition was not dependent on either calcium or substrate concentration. The altered fluorescence intensity and shifted negative bands of the circular dichroism spectrum suggest the direct interaction of the sinapic acid with the active site of the sPLA_2_-IIA enzyme. Furthermore, sinapic acid neutralized sPLA_2_-IIA induced erythrolysis, mouse paw edema and the hemorrhagic effect. As a result, sinapic acid is a potential therapeutic candidate for both inflammatory diseases and snakebite envenomation. However, more clinical studies are needed to claim sinapic acid as an anti-inflammatory drug.

## Figures and Tables

**Table 1:** The binding energy, H- bonds and hydrophobic interactions of genistein and sinapic acid with human sPLA_2_-IIA (PDB ID: 1POE).

No.	Ligands	Enzyme Name	Binding Energy (kcal/Mol)	No. of H Bonds	RMSD	Inhibition Constant (Ki) μM	Amino Acids Involved in Interactions Nteractions
1	Genistein	1POE (Human PLA_2_)	−7.2	02	80.96	1.95	Val45, Asp48, Cys49, Lys52, Thr121, Cys124
2	Sinapic acid		−7.6	01	83.44	2.71	Gly31, Gly32, Asp48, Cys49, Lys52, Thr121, Pro122, Cys124,

**Table 2:** The antioxidant activity of sinapic acid.

Antioxidant Assays	Sinapic Acid (25 µM)	Ascorbic Acid (25 µM)	Quercetin (25 µM)	α-Lipoic Acid (25 µM)
DPPH free radical	89% ± 2.13	96% ± 1.78	NT	NT
Reducing power	63.5% ± 2.05	NT	66.5% ± 1.82	NT
Anti-lipid peroxidation	80.6% ± 1.07	NT	NT	89% ± 1.69

NT—not tested.

**Table 3:** IC_50_ value of sinapic acid for sPLA_2_-IIA enzyme.

Enzyme	Specific Activity * (nmol/mg/min at 37 °C)	IC_50_ (μM) ^#^		Type of Inhibition
		Sinapic Acid	Genistein	
sPLA_2_-IIA	160.0	4.16 ± 0.13	11.75 [58]	Both are competitive inhibitors bound to the active site of the sPLA_2_-IIA.

* The specific activity is defined as nmoles of fatty acid released/mg of protein/min at 37 °C. ^#^ IC_50_ value is the concentration that inhibits 50% sPLA_2_-IIA enzyme activity.

**Table 4:** Secondary structure of sPLA_2_-IIA enzyme upon addition of sinapic acid.

* Secondary Structure of sPLA_2_-IIA	sPLA_2_-IIA Alone	sPLA_2_-IIA + Sinapic Acid (IC_50_)
α-helix	43.66%	21.47%
β-strand	10.62%	31.65%
Random coil	45.72%	46.88%

* Secondary structure of sPLA_2_ IIA was calculated with K2D3.

## Data Availability

Data is contained within the article.
