# Correction: Giresha et al. Sinapic Acid Inhibits Group IIA Secretory Phospholipase A_2_ and Its Inflammatory Response in Mice. *Antioxidants* 2022, *11*, 1251

**DOI:** 10.3390/antiox14111370

**Published:** 2025-11-18

**Authors:** Aladahalli S. Giresha, Deepadarshan Urs, Sophiya Pundalik, Rajkumar S. Meti, Siddanakoppalu N. Pramod, Ballenahalli H. Supreetha, Madhusudana Somegowda, Kattepura K. Dharmappa, Ahmed M. El-Shehawi, Sarah Albogami, Mona M. Elseehy, Abdullah Alaklabi, Hosam O. Elansary, Alanoud Omur A. Mehder, Eman A. Mahmoud

**Affiliations:** 1 Inflammation Research Laboratory, Department of Studies and Research in Biochemistry, Mangalore University, Jnana Kaveri Post Graduate Campus, Chikka Aluvara, Kodagu 571232, India; girishas25@mangaloreuniversity.ac.in (A.S.G.); deepdarshanurs@mangaloreuniversity.ac.in (D.U.); p.sophiya89@mangaloreuniversity.ac.in (S.P.); rsmeti@gmail.com (R.S.M.); suprithabh5985@gmail.com (B.H.S.); 2 Department of Biochemistry, School of Science, Jain (Deemed-to-Be University), JC Road, Bangalore 560027, India; 3 Department of Studies in Food Technology, Davangere University, Shivagangothri, Davangere 577007, India; pramodsn@davangereuniversity.ac.in; 4 Department of Plant Biochemistry, University of Agriculture and Horticulture Science, Shivamogga 577204, India; ysmadhu84@gmail.com; 5 Department of Biotechnology, College of Science, Taif University, Taif 21944, Saudi Arabia; a.elshehawi@tu.edu.sa (A.M.E.-S.); dr.sarah@tu.edu.sa (S.A.); 6 Department of Genetics, Faculty of Agriculture, University of Alexandria, Alexandria 21545, Egypt; monaahmedma@yahoo.com; 7 Department of Biology, Faculty of Science, University of Bisha, Bisha 61922, Saudi Arabia; alaklabia@gmail.com; 8 Plant Production Department, College of Food & Agriculture Sciences, King Saud University, Riyadh 11451, Saudi Arabia; 9 Family Education Department, Education College, Umm AL-Qura University, Mecca 24382, Saudi Arabia; aomehder@uqu.edu.sa; 10 Department of Food Industries, Faculty of Agriculture, Damietta University, Damietta 34511, Egypt; emanmail2005@yahoo.com

In the published paper [1] that there was a mistake in Figure 11a–c as the authors mistakenly uploaded hemorrhagic activity images during the manuscript submission.

The authors apologize for any inconvenience caused by this oversight. The new Figure 11 appears below.

Moreover, the title should be “Sinapic Acid” instead of “Sinapicacid”.

The authors state that the scientific conclusions are unaffected. This correction was approved by the Academic Editor. The original publication has also been updated.

## Figures and Tables

**Figure 11:**
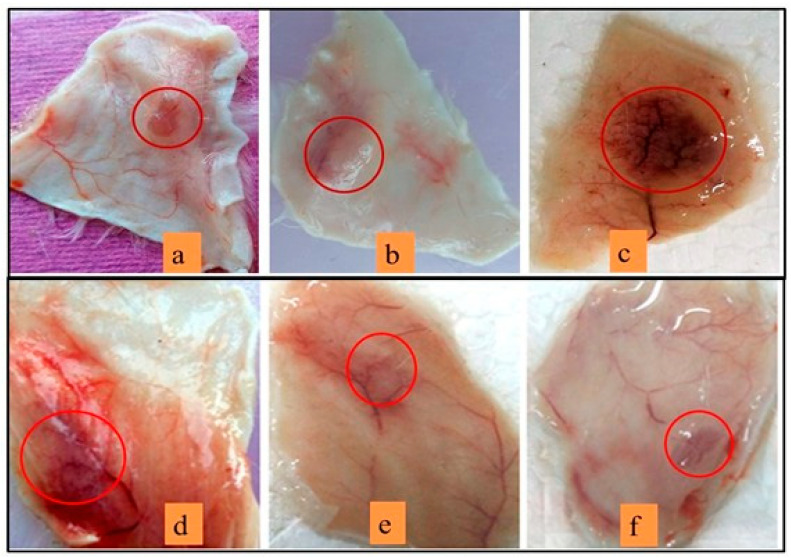
ReducingVR-HC-I mediated hemorrhagic activity by sinapic acid:Mice injected with 10 µg of sPLA_2_-IIA (**a**), 10 µg of VNTx-II alone (**b**), 10 µg of VR-HC-I (**c**). The injection of preincubated 10 µg of VR-HC-I with sinapic acid of 5 µM (**d**), 10 µM (**e**), 15 µM (**f**). The mice were sacrificed after 3 h, and hemorrhagic spots were recorded using the graph sheet.
